
JOURNAL INFORMATION
==============================
NLM Title Abbreviation: Innov Surg Sci
Journal ID: Innov Surg Sci
NLM Journal ID: 101708165
Journal ID: iss

Innovative Surgical Sciences

EISSN: 2364-7485
Publisher: De Gruyter

ARTICLE INFORMATION
==============================
PMCID: PMC11917180
PMID: 40110105
DOI: 10.1515/iss-2025-2010
Article ID: iss-2025-2010
Article version: 1
Subjects: Article

Abstracts DGU

Electronic publication date: 2025 Mar 19
Publication date: 2025 Mar
Volume: 10
Issue: Suppl1
First page: 388
Last page: 396
Copyright: © 2025 by Walter de Gruyter Berlin/Boston
Copyright year: 2025
Copyright holder: De Gruyter
License: This work is licensed under the Creative Commons Attribution 4.0 International License.
License URL: https://creativecommons.org/licenses/by/4.0/

Page count: 9

==============================

Abstracts – DCK 2025 – 142nd Congress of the German Society of Surgery – Munich, March 26–28 • DOI 10.1515/iss-2025-2010
Innov Surg Sci 2025; 10 (Special Suppl 1): s388–s396

### Assessment of trabecular bone density in Hounsfield units in the lumbar native CT cross-sectional image for osteoporosis diagnosis and fracture risk determination by different examiners in comparison with QCT and CTXA

ID: 220

**Kategorie: DGU - Alterstraumatologie**

Julian Ramin Andresen[1], Guido Schröder[2], Thomas Haider[1], Claus Kullen[3], Christoph Alexander Kopetsch[3], Hans-Christof Schober[4], Reimer Andresen[3]

[1]*Medical University of Vienna, Department of Orthopaedics and Trauma Surgery, Division of Trauma Surgery, Vienna, Österreich*, [2]*Rostock University Medical Center, Department of Traumatology, Hand and Reconstructive Surgery, Rostock, Deutschland*, [3]*Westkuestenklinikum Heide, Academic Teaching Hospital of the Universities of Kiel, Luebeck und Hamburg, Institute for Diagnostic and Interventional Radiology / Neuroradiology, Heide, Deutschland*, [4]*OrthoCoast, Practice for Orthopedics and Osteology, Wolgast, Deutschland*

**Background:**
A loss of bone mineral density (BMD) leads to osteoporosis and osteoporotic vertebral fractures (OVF). The extent to which assessment of osteoporosis and fracture risk in the spine is possible through density determination in Hounsfield units (HU) should be examined in comparison with QCT and CTXA data. The next question was whether quantitative BMD values could be calculated from the HU values.

**Materials and methods:**
A total of 240 patients (pat.) were included in the study, comprising 200 women with a mean age of 65.3 years (min: 24; max: 91) and a BMI of 27.1 kg/m2 (min: 17.7; max: 60.6), and 40 men with a mean age of 68.5 years (min: 42; max: 84) and a BMI of 24.9 kg/m2 (min: 17.6; max: 33.8), all being investigated for osteoporosis. Pat. were recruited from the outpatient departments of bariatric surgery, gynecology, geriatrics, neurosurgery, orthopedics, and traumatology. BMD in mg/cm3 was determined using QCT at the level of L1, L2, and L3 vertebrae, with adjacent vertebra considered in case of vertebral deformity. Following anonymization, three experienced radiologists performed additional measurements of trabecular density in HU in the same vertebrae (a total of 720 vertebral bodies). This was done by manually positioning an ellipsoidal region of interest (ROI) in the mid-vertebral trabecular space on sagittal reformatted CT images. Additionally, BMD assessment of the hip in mg/cm2 was conducted using CTXA. Osteoporotic vertebral fractures (OVF) were detected and classified on lateral X-rays of the thoracic and lumbar spine. MRI examinations were included in cases of suspected sacral insufficiency fractures.

**Results:**
For the lumbar spine, the median HU was 89.9 (67.9–126.9) and the median BMD was 73.7 (57.1–104.2) mg/cm3. With a correlation of $R2 = 0.98$ ($p < 0.001$), HU values can be converted into quantitative values in mg/cm3 using the following formula: XqSpine = 0.84+(0.81×HU). Trabecular density values < 100 HU serve as a threshold for osteoporosis. HU values < 79.8 and BMD < 65.5 mg/cm3 were significantly associated with increased OVF. Among 103 out of 240 pat., at least one OVF was detected, with no fractures observed cranially to T5. Additionally, sacral fractures were found in 32 out of 103 pat., who exhibited the lowest density values with a median HU of 31.8 (12.7–58.2). Comparable HU values were determined regardless of the examiners, with no significant difference found ($p > 0.05$). CTXA showed a correlation with lumbar spine HU values of $R2 = 0.69$ ($p = 0.238$).

**Conclusion:**
An opportunistic evaluation based solely on HU values seems validly feasible in the lumbar native CT cross-sectional image, with experienced examiners yielding comparable results. Density measurements in HU values can be converted into quantitative BMD values in mg/cm³, allowing for a good estimation of osteoporosis and fracture risk determination.

Open Access. © 2025 The Author(s), published by De Gruyter. This work is licensed under the Creative Commons Attribution 4.0 International License.

**ATZ DGU® - Geriatric co-management across Germany**
ID: 253

**Kategorie: DGU - Alterstraumatologie**

Charlotte Ramadan[1], Jeanette Köppe[2], Janette Iking[1], Karen Fischhuber[2], Markus Blätzinger[3], Josef Stolberg-Stolberg[1], Michael J. Raschke[1], J. Christoph Katthagen[1]

[1]*Klinik für Unfall-, Hand- und Wiederherstellungschirurgie, Münster, Deutschland*, [2]*Institut für Biometrie und Klinische Forschung, Münster, Deutschland*, [3]*AUC - Akademie der Unfallchirurgie, München, Deutschland*

**Background:**
A decade ago, the German Trauma Society (DGU) initiated the AltersTraumaZentrum (ATZ) certification DGU® to face the interdisciplinary challenges of ortho-geriatric co-management, a topic of increasing relevance regarding demographic change. The aim of this study was to illustrate various characteristics of ATZs – such as interdisciplinary cooperations, the level of trauma care and their distribution across the German states including hospital and population data.

**Materials and methods:**
ATZs validly certified by June 2023 were assigned to their level of trauma care (from level 3 to 1 trauma centers), these data were provided by the German Academy of Trauma Surgery (AUC). For allocation, institutional numbers gathered in publications of the National Association of Statutory Health Insurance Funds published in 2020 were used. Moreover, correlations with several parameters comparing the German states were included (Statistisches Bundesamt, as of 2023) and analyzed using Spearman correlation coefficient.

**Results:**
Due to cooperations, 191 institutions formed 147 ATZs, with 20 hospitals contributing geriatric expertise only which were excluded from further calculations, 16 external cooperating trauma surgery departments and 155 institutions providing both specialties. Among these 155, 43 held a cooperative certification and thus accounted for 73% of cooperating ATZ hospitals (n = 59).
Out of the certified facilities with trauma surgery departments (n = 171), 31% were level 3, 32% level 2 and 23% level 1 trauma centers providing the highest level of care, while 14% had none. Non cooperative ATZ hospitals were mostly level 2 trauma centers (34% > level 3 = 32% > level 1 = 22% > none = 12%). In contrast, cooperatively certified institutions (34.5%) were often level 2 or 3 trauma centers (both 29% > level 1 = 24% > none = 19%).
The number of ATZs per German state was correlated with the number of inhabitants ($r_s$ = 0.92), hospitals ($r_s$ = 0.88) and cases in total in 2022 ($r_s$ = 0.92) ($p < 0.001$ each) but not with density of population or the amount of elderly inhabitants older than 65 years ($p > 0.05$).
Most ATZ institutions were located in North Rhine-Westphalia (64) and Bavaria (29), while there was only one in Bremen as well as Saarland and none in Mecklenburg-Western Pomerania. Only 18 of 171 surgical ATZ institutions were located in the eastern part of Germany, three of them in Berlin, although on average, population there was older. Still, data for these states differed clearly when population size was taken into account, for example Saxony-Anhalt showed nearly three times as many ATZ hospitals per million than Saxony or Brandenburg.

**Conclusion:**
The distribution of levels of trauma care referred to the main objectives associated with the two certificates; trauma surgery on the one hand, geriatric co-treatment on the other. Some states located in eastern parts of Germany seemed to be disadvantaged regarding their amount of ATZs and proportion of elderly inhabitants. Whether this is associated with adverse effects on patient outcome is subject to further investigation.

### Minor trauma in nursing home residents- Identifying facilitators and barriers of telemedical surgical assessments in geriatric patients to reduce nursing-home sensitive conditions. Data and experiences from the Optimal@NRW study.

ID: 464

**Kategorie: DGU - Alterstraumatologie**

Miriam Katharina Hertwig[1], Jenny Unterkofler[2], Klemens Horst[1], Frank Hildebrand[1], Jörg Christian Brokmann[3]

[1]*Uniklinik RWTH Aachen, Klinik für Orthopädie, Unfall- und Wiederherstellungschirurgie, Aachen, Deutschland*, [2]*Uniklinik RWTH Aachen, Klinik für Anästhesiologie, Aachen, Deutschland*, [3]*Uniklinik RWTH Aachen, Zentrum für klinische Akut- und Notfallmedizin, Aachen, Deutschland*

**Background:**

Recently, the German healthcare system is confronted with diverse challenges, which are caused, among other reasons, by an increasing number of older adults, higher incidents of chronic conditions, and the lack of medical professionals. One effect of those changes are so-called nursing-home-sensitive conditions (NHSCs): nursing home residents (NHR) are often transported to hospitals with diseases that could have been treated outpatient if a suitable service was available [1]. Those NHSCs are associated with the financial burden for the system and health-related complications for the residents (like increased risk for delirium) [1,2]. For trauma surgeons in emergency rooms, this is a familiar situation: the rescue service presents an NHR in the middle of the night who fell and needs to be examined for possible consequences of the trauma. But aren't there any new delivery models with the potential to reduce NHSC and organize trauma examinations differently? We hypothesized that telemedicine is an option in minor trauma cases and that physical examination can be realized as a delegable action under professional telemedical supervision.

**Materials and methods:**

During a cluster-randomized trial (Optimal@NRW), 24 nursing homes in the Aachen region of western Germany were supplied with a 24/7 telemedical consultation service with video and audio connection. The delivery model also included real-time vital data monitoring, an electronic stethoscope, and the opportunity to send out a non-physician assistant to perform point-of-care diagnostics and delegable actions. A retrospective analysis of the performed telemedical consultations examined the minor trauma cases regarding reasons for presentation, diagnostics, and treatment to conclude on facilitators and barriers of telemedical surgical assessment.

**Results:**

During the trial, 338 teleconsultations were performed, of which 29 cases complied with the definition of minor trauma (defined by ICD-10 groups and predefined tracer diagnosis). The median age was 84, and 46.1% had care level 4. Most cases (82%) were teleconsultated because of acute falls or consequences of falls (focus on head and upper extremity injuries). Four cases (13,8%) were follow-up checks of patients to ensure timely recognition of new symptoms. In all cases, the caregiver performed a physical examination under the telephysician's supervision. In 72.4%, vital data were raised. In 10%, a mobile assistant was sent out for delegable actions. The telephysician prescribed pain medication in 24% of the cases; in 23%, a wound dressing was arranged. In 4 cases (13.7%), the telephysicians indicated the need for transport to the emergency room: three due to a head trauma in combination with oral anticoagulation and one for exclusion of a fracture.

**Conclusion:**
As 86.3% of the minor trauma cases were treated ambulatory, transports to hospitals could be circumvented, which offers a new perspective on minor trauma care in NHR. The physical examination can be a delegable action, especially under video-driven supervision. The active participation of the residents in demonstrating a range of movements and diligent caregivers facilitated telemedical surgical examinations. The possibility of having a live view of vital data also facilitated the surgical assessment. Barriers were trauma in patients with oral anticoagulation or those who recently had surgery, as those required a mandatory x-ray or CT scan. Besides, telemedicine for NHR is an effective way to perform timely examinations with fewer medical personnel involved, like hospital transport, and should be considered for NHR without oral anticoagulation.

[1] Valk-Draad, M. P. et al., Pflegeheim-sensitive Krankenhausfälle und Ansätze zur Verringerung der Hospitalisierung von Pflegeheimbewohnerinnen und -bewohnern. Bundesgesundheitsblatt - Gesundheitsforschung - Gesundheitsschutz, 2023. 66(2), p. 199–211.
[2] Kurte, M. S. and K. Blankart (2019). Ambulant-sensitive Krankenhausfälle in Deutschland – Abgrenzung, Prävalenz und Kosten. Gesundheitsökonomie & Qualitätsmanagement 24(6): 277-291.

**How does the level of care change after proximal humeral fracture in older people? – A propensity score matching analysis comparing surgical and non-operative treatment**
ID: 604

**Kategorie: DGU - Alterstraumatologie**

Jeanette Köppe[1], Josef Stolberg-Stolberg[2], Janette Iking[2], Karen Fischhuber[1], Michael Raschke[2], Jan Christoph Katthagen[2]

[1]*Universität Münster, Institut für Biometrie und Klinische Forschung, Münster, Deutschland*, [2]*Universitätsklinikum Münster, Klinik und Poliklinik für Unfall-, Hand- und Wiederherstellungschiurgie, Münster, Deutschland*

**Background:**
The proximal humeral fracture (PHF) is one of the most common fractures in older people. The extent to which a PHF can limit independence and thus also contribute to an increase in the level of care (LoC) has not yet been systematically investigated. The aim of this study was to analyze the need for care after PHF and to investigate if differences between treatment methods can be observed or not.

**Materials and methods:**
Retrospective insurance data of the BARMER health insurance company were analyzed. All patients (age above 65 years) with an in- or outpatient coded PHF (ICD S42.2) between 01/2017 and 09/2022 were included to the study. Treatment allocation was performed during the first 21 days after fracture and patients with shorter follow-up time were excluded (N=1,203). With a median follow-up of 37.5 months, patients were observed from final treatment allocation (i.e. 21 days after PHF) to the end of follow-up (given by end of the study 31-12-2022, exit from database, or death from any cause).
Patients were grouped in non-operative and surgical treatment (locked plate fixation for simple and multi-fragmented fractures: sLPF/LPF; OPS 5-793.k1, 5-793.31; 5-794.21, 5-794.k1; reverse total shoulder arthroplasty: RTSA; OPS 5-824.21 and other surgical treatment). Pre-existing LoC of assisted living was detected within two years before PHF, whereby the latest level was used. To account for differences between non-operative and surgical treatment, a 1:1 propensity score matching including age, sex, pre-existing LoC, year of fracture and patients risk profile was performed. Overall survival (OS), worsening of the LoC and the need of nursing home care (NHC) were defined as the primary endpoints of the study. For all endpoints, the cumulative incidence function (event rate) were estimated using Aalen-Johansen estimate and differences between treatment groups were analyzed using multivariable Fine and Gray Cox regression model. P-value adjustment was performed using Bonferroni-Holm method account for the multiple test problem.

**Results:**
In total, 54,595 patients (median age 79 years, 84% female sex) were included to the study. Surgical treatment was performed in 44.3% of all patients, with RTSA being the most frequent treatment strategy (sLPF: 5.9%, LPF 30.9%, RTSA 32.2%, others 13.8%). Surgically treated patients had less often pre-existing LoC. After 1:1 propensity score matching, N=30.838 were included for further analyses.
One year after PHF, 21.3% of the patients had a higher LoC than before PHF. After multivariable regression, patient treated with RTSA showed significantly higher risk for a worsening of the LoC compared to the non-operative treatment cohort (HR 1.23; 95%CI 1.17 – 1.33; adjusted p<0.001), on the one hand. On the other hand, patients treated with sLPF/LPF compared to non-operatively treated patients showed no differences (adjusted p=0.593). However, differences between patients treated with RTSA and patients with non-operative treatment were only observed for a low increase of the LoC, i.e. transformations from no LoC to LoC I (HR 1.40; 95%CI 1.27 – 1.53; p<0.001) and achieving a LoC II (HR 1.31; 95%CI 1.19 – 1.44; p<0.001). Apart from this, no differences in the achievement of severe impairment (i.e. LoC ≥ III) were observed between both cohorts.
A total of 7.4% of patients (before matching) had already been cared in a nursing home before the PHF. These patients were less often treated surgically (patients from NCH 29.0% vs patients living at home 45.6%). During the course of follow-up, the proportion of patients (matched cohort) cared in a nursing home increases relevantly: 11.0% of patients, who previously lived at home, were cared in a nursing home one year after PHF. However, no differences in the need of a NHC after PHF between surgically and non-operatively treated patients were observed (p>0.999).

**Conclusion:**
PHF has a decisive influence on the independence and LoC of older people. This study was the first to systematically describe this impact. The increase in the level of care is not only associated with a reduction in the patient's quality of life, but also with an enormous financial burden on our healthcare system. Individual, risk-adapted treatment of PHF is therefore a worthwhile goal for the coming years.

### Systemic metal pollutant exposure after ESIN osteosynthesis of paediatric forearm fractures

ID: 532

**Kategorie: DGU - Kindertraumatologie**

Ahmed Natsha[1], Anne Mittenmayer[1], Mohamad Alrefai[1], Matthias Nissen[1], Joachim Kübler[2], Jochen Hubertus[1]

[1]*Kindermedizinisches Zentrum der St. Elisabeth Gruppe - Klinik für Kinderchirurgie, Witten, Deutschland*, [2]*Klinik für Kinderchirurgie und Kinderurologie im Eltern-Kind-Zentrum Prof. Hess am Klinikum Bremen-Mitte, Bremen, Deutschland*

**Background:**
Elastic stable intramedullary nailing (ESIN) is often used for treatment of paediatric forearm fractures. It is known from studies on adults that metal implants can lead to systemically detectable release of metal into the body. However, there is little data available for children. Knowledge is limited to a few studies on curved metal bar implants for the correction of funnel breasts or implants for stabilization surgery of deformed spines, where systemic detection of metal in the blood and urine has been demonstrated in children. Notably, these implants had remained in the body for several years. In this pilot study, we want to quantify the possible contaminant load of chromium and nickel in the urine and blood of children before and after ESIN implantation. Our hypothesis is that, according to the intramedullary location of the metal, and also on the basis of data from other studies, metal residues can be detected even after relatively short periods of about a few weeks to months.

**Materials and methods:**
This prospective monocentric pilot study (DRKS ID: 00034133) attempts to quantify the contamination of nickel and chromium in the blood and urine before and after metal ESIN implantation in the medullary canal of forearm bones in 22 children. The primary endpoints are nickel and chromium levels in urine and serum at two different timepoints. Secondary endpoints are parameters such as body weight, length, number of implanted ESIN, and implantation duration.

**Results:**
Since May 2024, we are recruiting patients with forearm shaft fractures necessitating ESIN implantation. Upon ESIN implantation, a blood and urine sample is taken in order to determine the baseline control value (metal pollutant-free control) which is compared with a second sample taken during surgery for removal of ESIN (maximum systemic metal burden). Further secondary endpoints should then also be correlated with the values collected.

**Conclusion:**
The results will be presented at the congress with possible implications on the use of metal ESIN in treatment of paediatric forearm bone fractures.

**Prehospital triage algorithms for mass casualty incidents in Germany - Accuracy of the PRIOR® pre-screening algorithm based on patient data from the TraumaRegister® DGU**
ID: 652

**Kategorie: DGU - Versorgung im Katastrophen-, Einsatz und Terrorfall**

Miriam Katharina Hertwig[1], Dan Bieler[2], Klemens Horst[1], Frank Hildebrand[1], Rolf Lefering[3]

[1]*Uniklinik RWTH Aachen, Klinik für Orthopädie, Unfall- und Wiederherstellungschirurgie, Aachen, Deutschland*, [2]*Bundeswehrzentralkrankenhaus Koblenz, Koblenz, Deutschland*, [3]*Institut für Forschung in der Operativen Medizin (IFOM), Köln, Deutschland*

**Background:**
The number of significant incidents has risen steadily over the last ten years. In Europe, 630 incidents were registered between 2012 and 2022, according to a data query at the international disaster database EM-DAT (Centre for Research on the Epidemiology of Disasters). Natural disasters like floods, but also those with a terrorist background, mass panic, or accidents in densely populated areas are leading to so-called mass casualty incidents (MCI). This situation is defined as the presence of more injured persons than the capacities of the regular rescue service can handle [1]. Professional emergency services, which are the first on the scene, play an essential role as they need to decide the treatment priority of the injured. A vital aspect of triage is the assignment to a triage category. The triage categories are intended to help ensure that patients with severe injuries receive treatment as quickly as possible by downgrading treatment priority for those with minor injuries. Several established triage algorithms are in use internationally, for example, START, Sieve Triage, and SALT. In Germany, however, death as one triage category can legally only be determined by a doctor, which limits the use of international triage methods in this country [2]. Therefore, a separate procedure for pre-triage by rescue teams was developed in 2014 by a cooperation between the Federal Office of Civil Protection and Disaster Assistance and the German Society for Disaster Medicine: the PRIOR® algorithm. In contrast to Anglo-American algorithms, PRIOR® does not contain any categories with expected death or death. The procedure has not yet been sufficiently validated in trials, cohort, or registry studies. Therefore, this study aims to evaluate the PRIOR® triage algorithm for its mapping accuracy using the German TraumaRegister DGU®.

**Materials and methods:**
Data extraction was performed after receiving permission from the TraumaRegister DGU® of the German Trauma Society. The TraumaRegister DGU® was founded in 1993 and is a multi-center database with pseudonymized and standardized documentation of severely injured patients. Currently, more than 28,000 cases from almost 700 hospitals are entered into the database per year. Data from 2017-2022 were used for analysis in the study. After data clearance, the remaining data points were assigned the triage categories of PRIOR®. Each category was then analyzed further regarding mortality in hospital and 24-hour mortality to conclude its accuracy.

**Results:**
Data from 21.164 cases were assigned PRIOR® Category 1 (red; immediate treatment), 13.934 cases were assigned to Category 2 (yellow; fast treatment), and 44.727 cases were assigned to Category 3 (green; no urge). 24h mortality in patients assigned category 1 varied from 5.6% to 29.6% depending on the leading problem, disturbance of consciousness being the most relevant, leading to high mortality. Cases assigned to category 2 showed a mortality rate of 9,4% but 24h mortality of 4.7%. Those mortality rates implicate a high accuracy of PRIOR in categories 1 and 2. Cases in category 3 showed mortality in hospitals of 1,4% and 0.3% in 24-hour mortality, which also seems accurate. A decent analysis of these fatalities showed that 66% of the people assigned to Category 3 who died in the hospital were 80 years or older.

**Conclusion:**
While the PRIOR® algorithm shows promise, our study also highlights the need for further research and refinement. There might be an underestimation of injury severity in older people in the algorithm. This is in line with international studies that have already recognized higher mortality rates in older people after triage in the emergency room [3]. There are approaches to assign older people to a higher triage category to improve accuracy and reduce 24h mortality [4]. Due to our demographic change, those findings should be considered for triage algorithms. The analysis has limitations as the registry does entail only trauma cases and not another disease that might occur during an MCI. The lack of randomized controlled trials due to ethical issues only leaves simulations as an adequate method to examine triage accuracy of PRIOR®.

1. Bundesamt für Katastrophenschutz und Bevölkerungshilfe (BBK), BBK Glossar, https://www.bbk.bund.de/SharedDocs/Glossareintraege/DE/M/MANV.html
2. Gabriel, P., Huckenbeck, W. Todesfeststellung: Grundregeln, Durchführung und häufige Fehler. Z Allg Med 85, 460–465 (2009). https://doi.org/10.3238/zfa.2009.0460
3. Ginsburg AD, Oliveira J E Silva L, Mullan A, Mhayamaguru KM, Bower S, Jeffery MM, Bellolio F. Should age be incorporated into the adult triage algorithm in the emergency department? Am J Emerg Med. 2021 Aug;46:508-514. doi: 10.1016/j.ajem.2020.10.075. Epub 2020 Nov 4. PMID: 33191046.
4. Kemp, K., Alakare, J., Kätkä, M. et al. Effect of age adjustment on two triage methods. BMC Emerg Med 22, 52 (2022). https://doi.org/10.1186/s12873-022-00600-0
